# Distrontium lithium beryllium triborate, Sr_2_LiBeB_3_O_8_

**DOI:** 10.1107/S1600536812015164

**Published:** 2012-04-18

**Authors:** Na Yu, Ning Ye

**Affiliations:** a Fujian Institute of Research on the Structure of Matter, Chinese Academy of Sciences, Fuzhou, Fujian 350002, People’s Republic of China

## Abstract

Single crystals of distrontium lithium beryllium triborate, Sr_2_LiBeB_3_O_8_, were obtained by spontaneous nucleation from a high-temperature melt. In the Sr_2_Li[BeB_3_O_8_] structure, [BeB_2_O_7_]^6−^ rings, made up from one BeO_4_ tetra­hedron and two BO_3_ triangles, are connected to each other by [BO_3_] triangles to form the smallest repeat unit {[BeB_3_O_8_]^8−^} and then form chains along the *b* axis. The Sr^2+^ cations are seven- or eight-­coordinated and Li^+^ cations are tetra-­coordinated and lie between the chains.

## Related literature

Non-linear optical (NLO) applications of borate crystals with trigonal BO_3_ anions have been discussed by Chen *et al.* (1999 ▶). Among this group of compounds, beryllium borates are reported to exhibit the shortest transmission cut-off wavelength (Li, 1989 ▶). A review of the geometry of the BO_3_ group is given by Zobetz (1982 ▶), and a similar configuration of the [BeB_2_O_7_]^6−^ unit is found in LiB_3_O_5_ (LBO; Chen *et al.*, 2005 ▶) in which [B_3_O_7_]^5−^ rings are present. The structure of the beryllium borate group [BeB_2_O_7_]^6−^ is given by Li & Ye (2007 ▶).

## Experimental

### 

#### Crystal data

Sr_2_LiBeB_3_O_8_
*M* *_r_* = 351.62
Monoclinic, 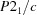

*a* = 8.609 (5) Å
*b* = 6.486 (4) Å
*c* = 12.868 (8) Å
β = 106.91 (1)°
*V* = 687.4 (7) Å^3^
*Z* = 4
Mo *K*α radiation
μ = 15.53 mm^−1^
*T* = 293 K
0.20 × 0.12 × 0.10 mm

#### Data collection

Rigaku Mercury2 diffractometer
Absorption correction: multi-scan (*CrystalClear*; Rigaku, 2007 ▶) *T*_min_ = 0.123, *T*_max_ = 0.212
5147 measured reflections
1574 independent reflections
1429 reflections with *I* > 2σ(*I*)
*R*_int_ = 0.038

#### Refinement

*R*[*F* ^2^ > 2σ(*F* ^2^)] = 0.026
*wR*(*F* ^2^) = 0.060
*S* = 1.05
1574 reflections
137 parameters
Δρ_max_ = 0.97 e Å^−3^
Δρ_min_ = −0.81 e Å^−3^

### 

Data collection: *CrystalClear* (Rigaku, 2007 ▶); cell refinement: *CrystalClear*; data reduction: *CrystalClear*; program(s) used to solve structure: *SHELXS97* (Sheldrick, 2008 ▶); program(s) used to refine structure: *SHELXL97* (Sheldrick, 2008 ▶); molecular graphics: *DIAMOND* (Brandenburg, 2004 ▶); software used to prepare material for publication: *SHELXTL* (Sheldrick, 2008 ▶).

## Supplementary Material

Crystal structure: contains datablock(s) global, I. DOI: 10.1107/S1600536812015164/br2197sup1.cif

Structure factors: contains datablock(s) I. DOI: 10.1107/S1600536812015164/br2197Isup2.hkl

Additional supplementary materials:  crystallographic information; 3D view; checkCIF report

## Figures and Tables

**Fig. 1.**
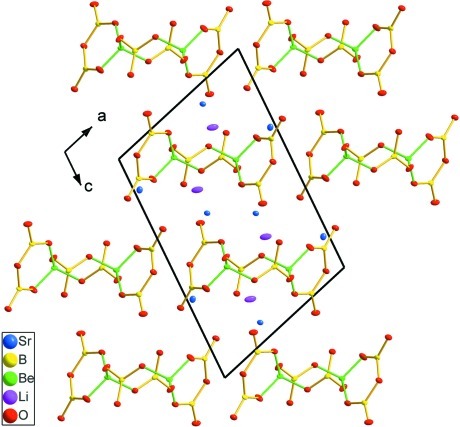
The crystal structure of Sr2Li[BeB3O8], viewed along the b axis, and drawn with anisotropic displacement paremeters at the 50% probability level. Sr—O bonds and Li—O bonds were omitted for clarity.

**Fig. 2.**
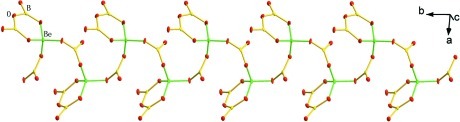
The parallel arrangement of the smallest repeat unit {[BeB3O8]8-} (n→∞) along b axis which forms a one-dimensional infinite chain.

**Fig. 3.**
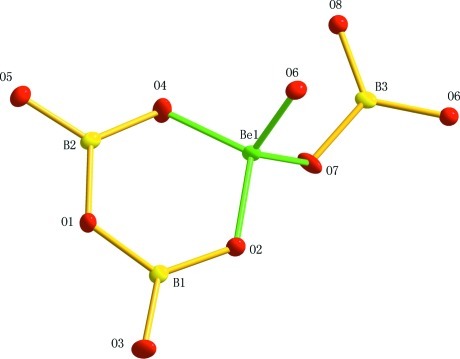
[BeB2O7]6- building unit in the title compound.

## supplementary crystallographic information

### Comment

Based on a theoretical study, beryllium borates possess the largest energy gap among all alkaline and alkaline earth borates, and hence the shortest transmission cut-off wavelength (Li, 1989). In addition, borate crystals containing parallelly aligned BO_3_ anionic groups are considered to be good candidates for NLO applications (Chen *et al.*, 1999). Therefore, beryllium borates are studied intensively with the purpose of searching for novel compounds with potential applications in the UV region. The title compound, Sr_2_LiBeB_3_O_8_, was found from the investigation of the beryllium borate system containing strontium and lithium.

A perspective view of the Sr_2_LiBeB_3_O_8_ structure in the a-c plane is shown in Fig.1. It contains a similar beryllium borate group [BeB_2_O_7_]^6-^ which was found in the structure Na_2_BeB_2_O_5_(Li & Ye, 2007) as the basic group in the Sr_2_LiBeB_3_O_8_ structure. In the structure of non-planar six-ring [BeB_2_O_7_]^6-^, the Be atoms are bonded to four O atoms to form distorted BeO_4_ tetrahedral. The B atoms are coordinated to three O atoms to form planar BO_3_ triangles and two planar BO_3_ groups share one common O1 atom, and each of them also shares a different O atom with a BeO_4_ tetrahedral.(Fig.3) This structure of the basic structural unit, [BeB_2_O_7_]^6-^, is similar to that of [B_3_O_7_]^5-^ in LiB_3_O_5_ (LBO) (Chen *et al.*, 2005), with a BO_4_ replaced by BeO_4_. In the Sr_2_LiBeB_3_O_8_ structure, the [BeB_2_O_7_]^6-^ rings are linked each other by a bridging BO_3_ group(B3 atom) to form the smallest repeat unit {[BeB_3_O_8_]^8-^} (n→∞) one dimensional chains along the b Axis (Fig.2). From the study of LBO, it is known that the [B_3_O_7_]^5-^ group can yield large NLO effects and short UV transmission cut-offs, but spatial arrangement of the endless helices of [B_3_O_7_](n→∞) chains along the *z* axis is unfavorable for producing a large birefringence. Therefore, the resulting layer structure of [BeB_2_O_7_](n→∞) along the *b* axis may be a good candidate for DUV NLO applications. Unfortunately, in the case of Sr_2_LiBeB_3_O_8_, the direction of [BeB_2_O_7_]^6-^ group along the *b* axis are completely opposite and, therefore, their contributions to the NLO effect cancel out.

### Experimental

Single crystals of Sr_2_LiBeB_3_O_8_ were grown from a high-temperature solution using SrO-B_2_O_3_—Li_2_CO_3_ as a flux. This solution was prepared in a platinum crucible after melting of a mixture of SrCO_3_, BeO, B_2_O_3_ and Li_2_CO_3_ at the ratio of SrO/BeO/B_2_O_3_/Li_2_CO_3_=4:2:3:2. The mixture (10 g) was heated in a temperature-programmable electric furnace at 1273 K until the melt became transparent and clear. The homogenized melt solution was then cooled rapidly (323 K/h) to the initial crystallization temperature (1073 K). It was further cooled slowly (276 K/h) to the final crystallization temperature (973 K) and then allowed to cool to room temperature after the furnace was turned off. The flux attached to the crystal was readily dissolved in water.

### Crystal data

**Table:**

Sr_2_LiBeB_3_O_8_	*F*(000) = 648
*M**_r_* = 351.62	*D*_x_ = 3.397 Mg m^−^^3^
Monoclinic, *P*2_1_/*c*	Mo *K*α radiation, λ = 0.71073 Å
Hall symbol: -P 2ybc	Cell parameters from 764 reflections
*a* = 8.609 (5) Å	θ = 4.1–27.5°
*b* = 6.486 (4) Å	µ = 15.53 mm^−^^1^
*c* = 12.868 (8) Å	*T* = 293 K
β = 106.91 (1)°	Prism, colorless
*V* = 687.4 (7) Å^3^	0.20 × 0.12 × 0.10 mm
*Z* = 4	

### Data collection

**Table:**

Rigaku Mercury2 diffractometer	1574 independent reflections
Radiation source: fine-focus sealed tube	1429 reflections with *I* > 2σ(*I*)
Graphite monochromator	*R*_int_ = 0.038
Detector resolution: 13.6612 pixels mm^-1^	θ_max_ = 27.5°, θ_min_ = 2.5°
CCD_Profile_fitting scans	*h* = −11→9
Absorption correction: multi-scan (*CrystalClear*; Rigaku, 2007)	*k* = −7→8
*T*_min_ = 0.123, *T*_max_ = 0.212	*l* = −16→16
5147 measured reflections	

### Refinement

**Table:**

Refinement on *F*^2^	Primary atom site location: structure-invariant direct methods
Least-squares matrix: full	Secondary atom site location: difference Fourier map
*R*[*F*^2^ > 2σ(*F*^2^)] = 0.026	*w* = 1/[σ^2^(*F*_o_^2^) + (0.0325*P*)^2^] where *P* = (*F*_o_^2^ + 2*F*_c_^2^)/3
*wR*(*F*^2^) = 0.060	(Δ/σ)_max_ < 0.001
*S* = 1.05	Δρ_max_ = 0.97 e Å^−^^3^
1574 reflections	Δρ_min_ = −0.81 e Å^−^^3^
137 parameters	Extinction correction: *SHELXL97* (Sheldrick, 2008), Fc^*^=kFc[1+0.001xFc^2^λ^3^/sin(2θ)]^-1/4^
0 restraints	Extinction coefficient: 0.0525 (14)

### Special details

**Table:**

Geometry. All e.s.d.'s (except the e.s.d. in the dihedral angle between two l.s. planes) are estimated using the full covariance matrix. The cell e.s.d.'s are taken into account individually in the estimation of e.s.d.'s in distances, angles and torsion angles; correlations between e.s.d.'s in cell parameters are only used when they are defined by crystal symmetry. An approximate (isotropic) treatment of cell e.s.d.'s is used for estimating e.s.d.'s involving l.s. planes.
Refinement. Refinement of *F*^2^ against ALL reflections. The weighted *R*-factor *wR* and goodness of fit *S* are based on *F*^2^, conventional *R*-factors *R* are based on *F*, with *F* set to zero for negative *F*^2^. The threshold expression of *F*^2^ > σ(*F*^2^) is used only for calculating *R*-factors(gt) *etc*. and is not relevant to the choice of reflections for refinement. *R*-factors based on *F*^2^ are statistically about twice as large as those based on *F*, and *R*- factors based on ALL data will be even larger.

### Fractional atomic coordinates and isotropic or equivalent isotropic displacement parameters (Å^2^)

**Table:**

	*x*	*y*	*z*	*U*_iso_*/*U*_eq_	
Sr1	0.35975 (4)	0.24425 (5)	0.42862 (2)	0.00870 (12)	
Sr2	0.96530 (4)	0.89027 (5)	0.34184 (2)	0.01077 (12)	
B1	0.7378 (5)	0.2697 (6)	0.4861 (3)	0.0081 (7)	
B2	0.9374 (5)	0.3471 (6)	0.3805 (3)	0.0086 (7)	
B3	0.4005 (5)	0.4529 (6)	0.2060 (3)	0.0089 (7)	
Be1	0.6756 (5)	0.5709 (7)	0.3439 (3)	0.0087 (9)	
Li1	0.3681 (9)	0.7734 (10)	0.3261 (5)	0.0212 (15)	
O1	0.8842 (3)	0.2319 (4)	0.4584 (2)	0.0115 (5)	
O2	0.6391 (3)	0.4284 (4)	0.43886 (18)	0.0108 (5)	
O3	0.7074 (3)	0.1423 (4)	0.56005 (19)	0.0135 (5)	
O4	0.8589 (3)	0.5215 (4)	0.3371 (2)	0.0143 (5)	
O5	1.0594 (3)	0.2620 (4)	0.3497 (2)	0.0118 (5)	
O6	0.5555 (3)	0.5273 (4)	0.22195 (18)	0.0105 (5)	
O7	0.6566 (3)	0.8100 (4)	0.37736 (19)	0.0132 (5)	
O8	0.3055 (3)	0.5106 (4)	0.26900 (19)	0.0114 (5)	

### Atomic displacement parameters (Å^2^)

**Table:**

	*U*^11^	*U*^22^	*U*^33^	*U*^12^	*U*^13^	*U*^23^
Sr1	0.01050 (19)	0.0059 (2)	0.01079 (19)	0.00014 (11)	0.00487 (13)	0.00049 (11)
Sr2	0.01267 (19)	0.00595 (19)	0.01254 (19)	−0.00039 (12)	0.00184 (13)	0.00046 (12)
B1	0.0104 (19)	0.0045 (19)	0.0099 (18)	−0.0021 (14)	0.0039 (14)	−0.0027 (14)
B2	0.0091 (18)	0.008 (2)	0.0102 (17)	−0.0036 (14)	0.0042 (14)	−0.0019 (15)
B3	0.0151 (19)	0.0043 (19)	0.0073 (17)	0.0016 (15)	0.0032 (14)	0.0022 (14)
Be1	0.012 (2)	0.005 (2)	0.010 (2)	−0.0002 (16)	0.0055 (16)	0.0020 (16)
Li1	0.039 (4)	0.010 (4)	0.021 (3)	−0.007 (3)	0.019 (3)	−0.005 (3)
O1	0.0113 (13)	0.0102 (14)	0.0147 (12)	0.0034 (9)	0.0063 (9)	0.0045 (10)
O2	0.0117 (12)	0.0087 (13)	0.0136 (12)	0.0004 (9)	0.0062 (9)	0.0027 (10)
O3	0.0227 (14)	0.0057 (13)	0.0154 (12)	0.0011 (10)	0.0109 (10)	0.0022 (10)
O4	0.0122 (13)	0.0085 (13)	0.0248 (13)	0.0011 (10)	0.0093 (10)	0.0070 (11)
O5	0.0127 (13)	0.0097 (14)	0.0148 (12)	0.0001 (9)	0.0069 (10)	−0.0013 (10)
O6	0.0123 (12)	0.0091 (13)	0.0112 (12)	−0.0013 (9)	0.0050 (9)	−0.0012 (10)
O7	0.0253 (14)	0.0039 (13)	0.0091 (11)	0.0031 (10)	0.0029 (10)	0.0009 (10)
O8	0.0126 (12)	0.0103 (14)	0.0125 (12)	−0.0018 (10)	0.0054 (9)	−0.0019 (10)

### Geometric parameters (Å, º)

**Table:**

Sr1—O5^i^	2.491 (3)	B1—O3	1.342 (4)
Sr1—O7^ii^	2.565 (3)	B1—O2	1.360 (5)
Sr1—O3^iii^	2.586 (3)	B1—O1	1.428 (5)
Sr1—O8	2.620 (3)	B2—O5	1.344 (4)
Sr1—O2	2.654 (3)	B2—O4	1.352 (5)
Sr1—O6^iv^	2.663 (3)	B2—O1	1.428 (4)
Sr1—O2^ii^	2.722 (3)	B3—O8	1.361 (5)
Sr1—O3	3.051 (3)	B3—O6	1.377 (5)
Sr2—O8^v^	2.478 (3)	B3—O7^iv^	1.395 (5)
Sr2—O5^vi^	2.536 (3)	Be1—O7	1.630 (5)
Sr2—O5^vii^	2.551 (3)	Be1—O6	1.634 (5)
Sr2—O4	2.556 (3)	Be1—O2	1.634 (5)
Sr2—O1^viii^	2.642 (3)	Be1—O4	1.638 (5)
Sr2—O3^viii^	2.740 (3)	Li1—O3^ii^	1.849 (7)
Sr2—O7	2.872 (3)	Li1—O8	1.872 (7)
Sr2—O1^vi^	2.873 (3)	Li1—O6^v^	1.940 (7)
Sr2—O4^vii^	3.217 (3)	Li1—O7	2.389 (8)
			
O5^i^—Sr1—O7^ii^	93.38 (9)	O8^v^—Sr2—O7	52.40 (8)
O5^i^—Sr1—O3^iii^	81.09 (8)	O5^vi^—Sr2—O7	117.75 (8)
O7^ii^—Sr1—O3^iii^	74.69 (8)	O5^vii^—Sr2—O7	105.89 (8)
O5^i^—Sr1—O8	73.89 (8)	O4—Sr2—O7	59.49 (8)
O7^ii^—Sr1—O8	143.67 (8)	O1^viii^—Sr2—O7	90.30 (8)
O3^iii^—Sr1—O8	133.75 (8)	O3^viii^—Sr2—O7	141.98 (7)
O5^i^—Sr1—O2	144.86 (8)	O8^v^—Sr2—O1^vi^	74.15 (8)
O7^ii^—Sr1—O2	108.59 (8)	O5^vi^—Sr2—O1^vi^	50.90 (8)
O3^iii^—Sr1—O2	130.36 (8)	O5^vii^—Sr2—O1^vi^	142.30 (8)
O8—Sr1—O2	72.22 (8)	O4—Sr2—O1^vi^	126.47 (8)
O5^i^—Sr1—O6^iv^	101.50 (8)	O1^viii^—Sr2—O1^vi^	81.27 (9)
O7^ii^—Sr1—O6^iv^	137.73 (9)	O3^viii^—Sr2—O1^vi^	101.09 (8)
O3^iii^—Sr1—O6^iv^	69.01 (8)	O7—Sr2—O1^vi^	71.36 (8)
O8—Sr1—O6^iv^	78.59 (9)	O8^v^—Sr2—O4^vii^	93.68 (8)
O2—Sr1—O6^iv^	80.66 (8)	O5^vi^—Sr2—O4^vii^	64.26 (7)
O5^i^—Sr1—O2^ii^	91.93 (8)	O5^vii^—Sr2—O4^vii^	47.06 (8)
O7^ii^—Sr1—O2^ii^	59.23 (8)	O4—Sr2—O4^vii^	118.07 (7)
O3^iii^—Sr1—O2^ii^	132.93 (8)	O1^viii^—Sr2—O4^vii^	125.21 (8)
O8—Sr1—O2^ii^	86.79 (9)	O3^viii^—Sr2—O4^vii^	73.47 (8)
O2—Sr1—O2^ii^	77.27 (8)	O7—Sr2—O4^vii^	144.26 (7)
O6^iv^—Sr1—O2^ii^	156.42 (7)	O1^vi^—Sr2—O4^vii^	113.95 (7)
O5^i^—Sr1—O3	166.09 (8)	O3—B1—O2	123.9 (3)
O7^ii^—Sr1—O3	75.73 (8)	O3—B1—O1	116.1 (3)
O3^iii^—Sr1—O3	87.60 (8)	O2—B1—O1	120.0 (3)
O8—Sr1—O3	120.00 (7)	O5—B2—O4	124.2 (3)
O2—Sr1—O3	48.81 (8)	O5—B2—O1	115.4 (3)
O6^iv^—Sr1—O3	81.75 (7)	O4—B2—O1	120.2 (3)
O2^ii^—Sr1—O3	89.86 (7)	O8—B3—O6	122.7 (3)
O8^v^—Sr2—O5^vi^	87.68 (9)	O8—B3—O7^iv^	120.2 (3)
O8^v^—Sr2—O5^vii^	75.33 (8)	O6—B3—O7^iv^	117.2 (3)
O5^vi^—Sr2—O5^vii^	106.52 (6)	O7—Be1—O6	109.5 (3)
O8^v^—Sr2—O4	90.61 (9)	O7—Be1—O2	106.6 (3)
O5^vi^—Sr2—O4	177.22 (8)	O6—Be1—O2	114.5 (3)
O5^vii^—Sr2—O4	75.13 (8)	O7—Be1—O4	111.9 (3)
O8^v^—Sr2—O1^viii^	140.12 (7)	O6—Be1—O4	105.4 (3)
O5^vi^—Sr2—O1^viii^	100.72 (8)	O2—Be1—O4	109.1 (3)
O5^vii^—Sr2—O1^viii^	136.21 (8)	O3^ii^—Li1—O8	116.9 (3)
O4—Sr2—O1^viii^	79.23 (8)	O3^ii^—Li1—O6^v^	103.4 (3)
O8^v^—Sr2—O3^viii^	163.40 (8)	O8—Li1—O6^v^	137.1 (4)
O5^vi^—Sr2—O3^viii^	77.37 (8)	O3^ii^—Li1—O7	109.2 (3)
O5^vii^—Sr2—O3^viii^	101.86 (8)	O8—Li1—O7	110.9 (3)
O4—Sr2—O3^viii^	104.60 (8)	O6^v^—Li1—O7	65.3 (2)
O1^viii^—Sr2—O3^viii^	51.76 (7)		

Symmetry codes: (i) *x*−1, *y*, *z*; (ii) −*x*+1, −*y*+1, −*z*+1; (iii) −*x*+1, −*y*, −*z*+1; (iv) −*x*+1, *y*−1/2, −*z*+1/2; (v) −*x*+1, *y*+1/2, −*z*+1/2; (vi) *x*, *y*+1, *z*; (vii) −*x*+2, *y*+1/2, −*z*+1/2; (viii) −*x*+2, −*y*+1, −*z*+1.
